# Quantitative analysis and characterization of floral volatiles, and the role of active compounds on the behavior of *Heortia vitessoides*

**DOI:** 10.3389/fpls.2024.1439087

**Published:** 2024-08-23

**Authors:** Chenyu Qian, Wenqi Xie, Zhongqi Su, Xiujun Wen, Tao Ma

**Affiliations:** College of Forestry and Landscape Architecture, South China Agricultural University, Guangzhou, China

**Keywords:** floral volatiles, Heortia vitessoides, HS-SPME, antennal response, olfactometer bioassay

## Abstract

This study explores the role of floral volatile organic compounds (FVOCs) in insect behavior, focusing on *Aquilaria sinensis* (AS), a valuable tropical plant threatened by *Heortia vitessoides* Moore. Despite *H. vitessoides*’ attraction to AS and non-host plants like *Elaeocarpus decipiens* (ED) and *Dalbergia odorifera* (DO), little is known about their chemical interactions. FVOCs from these plants were analyzed at 9:00 and 18:00 using GC×GC-QTOF-MS and HS-SPME. The results showed that ED exhibiting the highest concentration (92.340 ng/mg), followed by DO (75.167 ng/mg) and AS (64.450 ng/mg). Through GC-EAD and EAG, a total of 11 FVOC compounds with electrophysiological activates were identified. These compounds, except linalool, showed dose-dependent responses. Y-Tube bioassays confirmed phenylethyl alcohol or the mixture of EAD-active compounds produced positive chemotactic responses in both males and females. FVOCs have the potential to be used as a natural and sustainable alternative to chemical insecticides in pest control.

## 1 Introduction

*Aquilaria sinensis* (Lour.) Gilg (Myrtales: Thymelaeaceae), the principal source of agarwood, is an economically plant that is primarily cultivated in tropical areas, such as South China, Vietnam, India, Malaysia, *etc* (Chen et al., 2016; Qiao et al., 2012), which has a long history of application in traditional medicines, religious ceremonies, and incense industries (Wang et al., 2021). However, the cultivation of *A. sinensis* forests is seriously threatened by *Heortia vitessoides* Moore (Lepidoptera: Crambidae), a severe and notorious pest (Cheng et al., 2019). The larvae of *H. vitessoides* feed on the leaves, causing weakening, defoliation and eventual death of *A. sinensis* trees. The widespread use of chemical insecticides to control *H. vitessoides* infestations in agriculture has resulted in the development of resistance in these insects, leading to increased pesticide usage and severe environmental pollution. Chemical insecticides can enter water bodies, soil, and the atmosphere, affecting not only the target pests but also non-target organisms. Therefore, it is necessary to explore alternative strategies for *H. vitessoides* management that minimize the use of chemical insecticides and their associated environmental risks.

The floral fragrance is a blend of low-molecular-weight volatiles with various chemical functional groups, endogenously synthesized by plants (Dudareva and Negre, 2005). These volatiles serve as unique scent signals that attract specific visitor insects for successful reproduction and evolution (Zhang et al., 2022). Flowers emit aroma volatiles as chemical cues (Pichersky and Gershenzon, 2002), and offer nectar, pollen, or oil as rewards (Schiestl et al., 2011). Aroma volatiles also has adaptive roles as repellents and physiological protectors (Borg-Karlson et al., 1993; Kessler et al., 2008). Even plant species with different evolutionary histories might emit similar composition of floral scent when pollinated by the same moth species (Powers et al., 2020). Therefore, the temporal rhythms of nectar production and scent emission should be taken into consideration when investigating the chemical mechanism that flowers adapt to their pollinators (Balducci et al., 2020).

Headspace solid phase micro-extraction (HS-SPME) is a widely employed technique for extracting volatile organic compounds (VOCs) from various matrices (Rajhi et al., 2022; Oliveira et al., 2022; Muñoz-Redondo et al., 2022). HS-SPME is known for its simplicity and ease of use, requires minimal sample preparation, and the extraction process can be completed in a relatively short period of time (Marinaki et al., 2023). The method’s high sensitivity and reliability make it a valuable tool for the analysis of complex mixtures found in environmental and biological samples (Domínguez et al., 2017; Issa et al., 2020). In general, HS-SPME’s extraction efficiency is affected by experimental parameters such as sample amount, time and temperature of incubation, extraction, and desorption, which are therefore included in most of the optimization attempts (Muñoz-Redondo et al., 2020, 2022; Pati et al., 2021).

The pollinating *H. vitessoides* moths prefer *A. sinensis*, *Elaeocarpus decipiens* (Malvales: Elaeocarpaceae), and *Dalbergia odorifera* (Rosales: Fabaceae) as their primary and alternative hosts, respectively (**Supplementary Figure 1**), indicating that the attractance of flowers may be due to the present of some common FVOCs from these three plants. Yet, the extent of their attraction to these plants and the underlying mechanism are not fully understood. In this study, we aim to characterize the chemical composition of the floral fragrance emitted by these plants and assess their similarities and differences. The study also explored the temporal variations in volatile emissions in the morning and dusk. Simultaneously, the electroantennographic (EAG) and Y-tube olfactometer techniques were utilized to examine the antennal and behavioral reactions of *H. vitessoides* moths to the active compounds identified through gas chromatography-electroantennography detection (GC-EAD). By identifying FVOCs, it is possible to create a targeted approach to pest management that reduces the negative impact of chemical pesticides on the environment.

## 2 Materials and methods

### 2.1 Plant materials

The host plant *A. sinensis* (AS) flower was collected from Tianlu Lake Park (Guangzhou, China, 23°14′27″N, 113°24′48″E). Two non-host plants, *E. decipiens* (ED) and *D. odorifera* (DO) were harvested at South China Agricultural University (23°9′44″N, 113°21′21″E). The flower samples were all harvested in the early morning (9:00) and dusk (18:00), respectively. The phenotypic traits of the three flower species were shown in **Supplementary Table 1**.

### 2.2 Insect rear

The larvae in the fourth or fifth instar of *H. vitessoides* were also collected from Tianlu Lake Park. The larvae were maintained under environmental conditions of 26 ± 1°C and 80 ± 5% relative humidity (RH) under a 14:10 light: dark schedule. Fresh leaves of *A. sinensis* were added to feed larvae each day. The adults were kept in fine nylon mesh cages (50 × 50 × 50 cm) and supplied with 20% honey solution.

### 2.3 Chemicals

High-performance liquid chromatography (HPLC)-grade methanol was obtained from Baishi Chemical (Tianjin, China). Hexanal (>99%), benzaldehyde (>98%), 1-octen-3-ol (>96%), benzyl alcohol (>99%), heptanal (>96%), 3-octanol (>99%), nonanal (>98%), (*E*)-2-hexenal(>98%), and phenylacetaldehyde (>99%) were purchased from Cato Research Chemicals Inc (Oregon, U.S); Phenylethyl alcohol (≥98%), caryophyllene (>98%), geraniol (≥98%), linalool (>98%), nerol (≥97%), (*E*)-β-ocimene (>98%) were obtained from Macklin (Shanghai, China). A mixture of normal alkanes (C8-C25) was purchased from Sigma (St Louis, MO, USA).

### 2.4 Sample preparation

Flower samples were collected in liquid nitrogen and freeze-dried at -70°C (Alpha2-4 LD Plus; Christ, Osterode, German). Flowers were powdered and weighed into a 20-mL headspace vial (Agilent Technologies, Inc., Santa Clara, CA, USA). A 50/30 μm DVB/CAR/PDMS coated SPME fibers (Supelco-Aldrich, Bellefonte, PA, USA) was thermally cleaned and desorbed between samples. Extraction was performed at different temperatures and times considered for optimization in the next section. The SPME fiber was placed into the injector port at 250°C for 3 min. Each SPME process was repeated three times. Clean vials were used as controls. Quality control (QC) samples were created by mixing equal quantities of each type of sample (DO, AS, and ED flower collected at 9:00 and 18:00). The QC sample was used for validation of the SPME method. Each aliquot of flower sample was tested in triplicate.

### 2.5 Optimization of HS-SPME

The optimization of HS-SPME parameters was performed to ensure good chromatographic signals. The total peak area of extracted compounds was used to evaluate extraction efficiency. The optimization of the SPME parameters included low, medium, and high levels: sample weight (50, 125, and 200 mg), incubation temperature (30, 45, and 60°C), equilibration time (10, 20, and 30 min) and adsorption time (20, 40, and 60 min). Injections were made in triplicate.

### 2.6 GC × GC-QTOFMS

A comprehensive two-dimensional gas chromatography (GC×GC) system consisting of a GC gas chromatography (7890B, Agilent) and a quadrupole time-of-flight mass spectrometry (QTOFMS, 7250, Agilent) instrument was used. Samples were introduced by a split/splitless injector (SSL) system with a CTC autosampler (PAL RSI 120, Agilent). Helium carrier gas (99.999%) was set at a constant flow of 1 mL/min. The oven temperature was programmed from 50°C (held for 3 min) to 250°C at 5°C/min (held for 1 min), for a total running time of 44 min. A DB-Wax column (30 m × 250 μm, 0.25 μm film thickness; Agilent) and a DB-17 column (1.2 m × 180 μm, 0.18 μm film thickness; Agilent) were used for first and second-dimensional separation, respectively. A solid-state modulator (SSM 1800, J&X Technologies, Shanghai, China) was installed between the 1^st^ and 2^nd^ columns, and the cold zone temperature of the SSM was set at -50°C. The modulation period was 6 s. The transfer line temperature and ion source temperature of MS were 280°C and 200°C, respectively. Electron ionization (EI) mode was at 70 eV, and full-scan acquisition mode with a mass range of 40–450 m/z.

The raw data were processed with Canvas (J & X Technologies). The compounds were identified by comparing the gas chromatography-mass spectrometry spectra with standards, or tentatively identified by comparing mass spectrometry spectra with the data in the National Institute of Standards and Technology (NIST20) library. RI was calculated based on the retention times of *n*-alkanes (C_8_-C_25_) under the same conditions.

Quantification: The concentration of the identified major compounds was calculated by an external standard operating curves method. The standards were diluted respectively in methanol in a series of concentrations (0.5, 1, 5, 10, 50, and 100 μg/mL). For those components without corresponding standards, the curve of compounds that have similar chemical structures and the response signal were used to conduct semi-quantification (Zhou et al., 2020). The signal-to-noise ratio (S/N) of 3:1 and 10:1 was used for the LOD and LOQ, respectively. Precision was evaluated by calculating each QC sample’s percent relative standard deviation (RSD) (n=3). The repeatability was accepted no more than RSD of 15% (**Supplementary Table 2**).

### 2.7 GC-EAD analysis

Flower compounds to which *H. vitessoides* moths responded were identified on an Agilent 7820A GC system with an HP-5MS capillary column and an EAD and flame ionization detector (FID). A mixture of all the VOCs (1 μL) was injected in splitless mode with injector and detector temperatures at 250 and 300°C, respectively. The effluent between FID and EAD was split at a 1:1 ratio with a glass Y splitter (Agilent). The experimental details were described by Ma et al. (2015). In brief, antenna of male or female moths was mounted onto an antenna holder for the EAG probe (PRG-2) and exposed to the GC effluent introduced into a glass transfer tube (15 mm ID) with purified and humidified air flow (100 mL/min). The constant airstream was supplied continuously from the EAD to the *H. vitessoides* antennae by an air stimulus controller (CS-55) (Syntech). The antennal signal was recorded simultaneously with the FID signal and considered active when it elicited antennal responses at least 5 times.

### 2.8 EAG test

The EAG system used to analyze *H. vitessoides* antennae responses to compound mixtures was modified from previous method (Magsi et al., 2021). The system consisted of a MP-15 probe/micromanipulator, an IDAC-2 data acquisition interface box, and a CS-55 air stimulus controller. The antenna was mounted on fork-shaped metal electrodes and fixed with electrode gel (Spectra 360, Parker Laboratory Inc., Orange, NJ, USA). Chemicals were dissolved in methanol at three concentrations (1, 10, and 100 μg/μL), with methanol as a control. Chemicals were applied to filter paper strip (1 × 2 cm) and inserted into a glass Pasteur pipet (15 cm long) in a mixing tube with moistened airflow (100 mL/min). The signal was analyzed using EAG Pro software (Syntech, Germany). Stimuli were delivered by puffing the humidified air for 0.3 s. The average EAG values of each chemical component were compared to methanol before and after stimulation.

### 2.9 Olfactometry bioassays

Behavioral bioassays were conducted using a Y-olfactometer consisting of a base tube (20 cm long × 4 cm diam.) with two 15 cm arms at 70° angles. Odor sources consisted of a 1 cm^2^ piece of filter paper placed at the end of each arm. The test solutions were dissolved to the concentration of 10 μg/μL. The filter paper piece loaded with 10 μL of the corresponding standards was the test arm, and those loaded with 10 μL methanol were set as the control arm. Charcoal-filtered and moisten air was pumped into the system at 600 mL/min by using a gas sampling instrument. In each test, a single male or female moth was introduced into the base of the main olfactometer tube, and response to the corresponding stimulus was observed for 5 min. The observed behavior was recorded as follows: (1) standard choice or control choice (entering the respective arm for more than 2 cm and remaining for at least 30 s); (2) no choice (do not reach or visit any arm). Each solution tested thirty females or males. During the assays, the position of each olfactometer arm was shifted every five tests.

### 2.9 Statistical analyses

The orthogonal partial least squares discriminant analysis (OPLS-DA) was carried out with SIMCA 14.0 software (Umetrics, Umea, Sweden). Venn graph was conducted online (http://jvenn.toulouse.inra.fr). An Independent sample t-test was conducted to compare the electrophysiological response values of *H. vitessoides* male and female moths in the EAG test by SAS 9.4 software (SAS Institute Inc., Cary, NC, USA). The choices made by moths in the bioassays were analyzed by the chi-square goodness-of-fit test (SAS 9.4). Origin 2022 (OriginLab Corporation, Northampton, MA) and GraphPad Prism 9 (GraphPad Software) were used for data visualization.

## 3 Results and discussion

### 3.1 Optimization of the HS-SPME conditions

#### 3.1.1 Effect of sample amount

HS-SPME technology relies on a dynamic equilibrium process that balances analytes among the sample, headspace, and fiber coating (Ho et al., 2006). Therefore, the extraction efficiency depends on the amount of flower sample in the vial, which affects the ionic strength and vapor pressure of volatile compounds (Wei et al., 2020). Increasing the sample quantity from 50 mg to 125 mg doubled the efficiency of volatile compound extraction, but no further increase in extracted volatiles was observed with 200 mg (**Supplementary Figure 2A**). Additionally, the number of extracted compounds significantly decreased when the sample amount exceeded 200 mg. The results suggested that insufficient sample amounts might lead to low enrichment of trace compounds, while saturation of fiber sites prevented the collection of additional volatiles.

#### 3.1.2 Effect of equilibration temperature

Increasing the equilibration temperature from 30 to 45°C led to an increase in the peak area of total volatile, reaching the highest performance at 45°C. To preserve natural volatiles, 60°C was chosen as the maximum temperature, but a further increase from 45 to 60°C decreased the peak areas and peak numbers (**Supplementary Figure 2B**). Due to the high-energy barriers that bind analytes to the matrix, the adsorption of volatile components is a slow process (Liang et al., 2022). Consecutive heating improves diffusion coefficients and analyte volatility (Wei et al., 2020). However, the incubation temperature above 50 °C may lead to the Millard reaction and Strecker degradation, leading to the adsorption of large weights volatiles and the desorption of small molecular weight volatiles (Mu et al., 2021). Additionally, the release and enrichment of analytes on the fiber are antagonistic energy phases (Zhang and Pawliszyn, 1993), indicating that higher temperatures may promote analyte release but inhibit fiber adsorption. Thus, 45°C was deemed an appropriate incubation temperature for further experiments. Under optimal temperature conditions, the total amount of absorbed compounds is highest, and the variety of compounds is most comprehensive.

#### 3.1.3 Effect of equilibration and adsorption time

The SPME method combines extraction and pre-concentration into a single step, with equilibration and adsorption being dynamic processes that are closely related to the efficiency of HS-SPME (Piri-Moghadam et al., 2017). Equilibration time is critical for analyte release into the headspace and is typically necessary before extraction (Ma et al., 2013). Equilibrium time of 20 min and 30 min were tested and showed little difference, with 20 minutes selected as the optimal equilibrium time to balance efficiency and time cost (**Supplementary Figure 2C**). Subsequently, the extraction time was investigated. As shown in **Supplementary Figure 2D**, a maximum peak area and peak number was observed when the extraction time increased from 20 min to 40 min, followed by a decrease at 60 min due to the competitive adsorption of analytes on the fiber coating (Pawliszyn et al., 2010). Previous studies have suggested that longer extraction times do not significantly increase signal intensities (Pico et al., 2022). Therefore, 40 min was selected as the optimal adsorption time since longer extraction times do not continually facilitate extraction after the fiber is thoroughly occupied by volatile substances.

### 3.2 Volatile analysis

#### 3.2.1 Floral volatile composition

**Figure 1:**
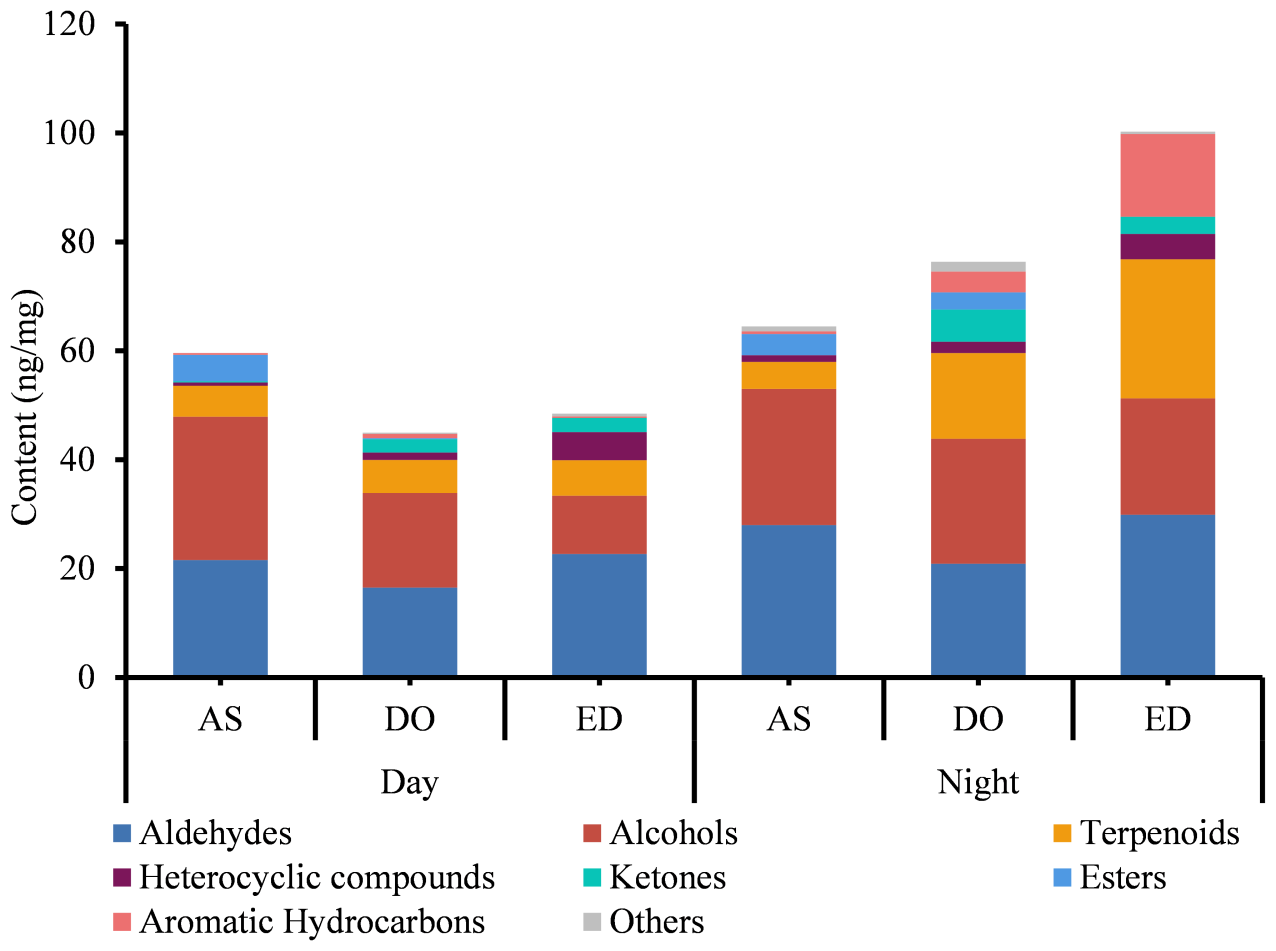
The analysis of VOCs of three tested flowers at day and night. DO, ED, and AS represent *Dalbergia odorifera*, *Elaeocarpus decipiens*, and *Aquilaria sinensis*, respectively.

The analysis of FVOCs revealed that the content of VOC varied among different flower cultivars, DO contained the most diverse range of compounds, whereas AS had the least. A total of 21 common FVOCs were identified. Additionally, the FVOCs were classified into various groups, including aldehydes, alcohols, terpenoids, *etc*. (**Supplementary Table 3**). Aldehydes and alcohols were the two most abundant groups in all three types of flowers, with concentrations ranging from 16.510 to 28.000 ng/mg and 10.724 to 26.321 ng/mg, respectively (**Figure 1**). Traces of esters (from 0 to 4.784 ng/mg), ketones (from 0 to 5.919 ng/mg) and heterocyclic compounds (from 0.033 to 3.091 ng/mg) were also detected. Hexanal was the most abundant VOC in DO flowers (from 7.135 ± 1.024 to 7.94 ± 0.182 ng/mg), while the concentration of 4-oxoisophorone was the highest in ED at 18:00 (11.26 ± 0.219 ng/mg). Furthermore, benzyl alcohol (ranging from 8.85 ± 0.141 to 10.277 ± 0.193 ng/mg) was the primary component of AS.

In accordance with the present results, these three plant species present pale petals and emit fragrant scents, providing a strong contrast to the darkness. Among those volatile compounds, linalool is a common component in floral aroma released by moth-pollinated flowers (Raguso and Pichersky, 1999; Knudsen et al., 2006), and has been shown to mediated a pollinator-plant association between the nocturnal moth *Manduca sexta* (Sphingidae) and *Datura wrightii* (Solanaceae) (Reisenman et al., 2010). (*E*)-α-bergamotene emitted from the night-flowering *Nicotiana attenuata* (Solanaceae) also had positive effects on *M. sexta* moth-mediated pollination success (Zhou et al., 2017), indicating that different host/non-host plants might use unique compound to attract the same pollinator. In *Asclepias speciosa* (Apocynaceae), phenylacetaldehyde was used alone to attract *Synanthedon myopaeformis* (Sesiidae) moths, which induced the most frequent probosci’s extension reflexes (Eby et al., 2013). The moth *Hadena bicruris* (Noctuidae) strongly preferred *Silene latifolia* (Caryophyllaceae) flowers over *S. dioica* (Bopp and Gottsberger, 2004), this preference could be attributed to the emission of lilac aldehydes and phenylacetaldehyde, which are known to be highly attractive to *H. bicruri* (Doetterl et al., 2009; Waelti et al., 2009). Thus, the VOCs profile of AS, ED, and DO may help explain the scent-mediated foraging behavior of *H. vitessoides* moths.

#### 3.2.2 Day-night comparisons of FVOCs profiles

The analysis of three flowers harvested at two sampling periods showed that the variety of compounds extracted from flowers harvested at 18:00 was higher compared to those harvested at 9:00 (**Figure 1**). At 18:00, a total of 56 compounds were found in DO and ED, followed by AS (45). Except for AS, the compound identified in DO and ED at 18:00 was higher than those found at 9:00. In addition, four unique compounds were detected in both ED and AS harvested at 18:00, while three unique components were identified in AS flower at 9:00 (**Figure 1**). Four aromatic hydrocarbons were specific to ED at 18:00, including m-ethylcumene (7.651 ± 0.629 ng/mg), pentamethylbenzene (0.487 ± 0.065 ng/mg), and 1,3-diethyl-4-methylbenzene (0.387 ± 0.005 ng/mg). Quantitatively, the concentration of compounds in the same flower species varied considerably between the two time slots. At 18:00, ED showed the highest concentration (92.340 ng/mg), followed by DO (75.167 ng/mg) and AS (64.450 ng/mg). While AS, ED, and DO samples at 9:00 were only with a total concentration of 59.610 ng/mg, 48.521 ng/mg, and 44.955 ng/mg, respectively.

FVOCs are crucial cues in habitats with low light availability (Klatt et al., 2013). The timing of floral scent emission was often linked to pollinators activity, with emissions intensifying when pollinators are active (Dötterl et al., 2012; Powers et al., 2020). For example, the scent composition of *Platanthera chlorantha* (Asparagales: Orchidaceae) differed between day and night, with (*E*)-β-ocimene and linalool being the dominant compounds detected in larger amounts before sunset and at night, but only in trace amounts during the day, which positively related with the visition of *Sphinx pinastri* (Lepidoptera: Sphingidae) (Steen et al., 2019). Similarly, the strongest volatile emission of *Silene otites* (Caryophyllaceae) was found immediately after sunset, characterized by attractants such as phenylacetaldehyde, 2-phenylethanol, and lilac aldehyde for some moths (Dötterl et al., 2012). Although the scent compounds in various flower stages seem to have no difference from the evening of anthesis to the following morning, the content for each compound might be varied (Yan et al., 2016). A potential benefit of temporally regulating aroma emission is to reduce the cost associated with the consumption of energetically expensive aroma (van der Niet et al., 2015). Based on our field and indoor observations, *H. vitessoides* moth exhibits increased emergence, foraging (flower visiting), and mating activities post-dusk, with peak activity occurring during this period. Therefore, quantitative analyses revealed that three plant species released more abundant FVOCs after 18:00, corresponding with previous studies that illustrated that plants were expected to emit a stronger floral scent at night when pollinators were most active (Powers et al., 2020).

### 3.4 Hierarchical clustering analysis

**Figure 2:**
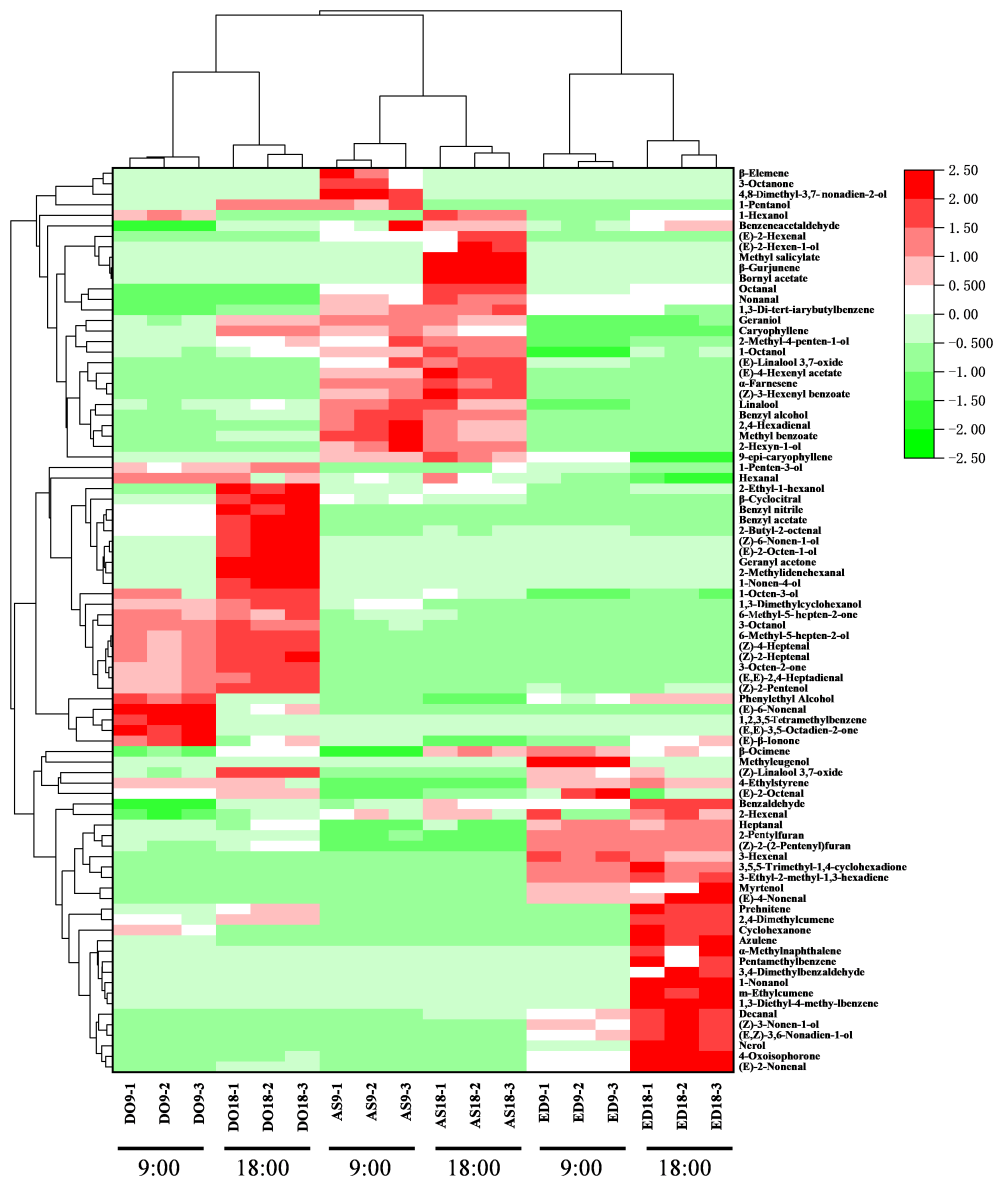
Hierarchical clustering and heatmap visualization of volatile compounds in three flower species. DO, ED, and AS represent *Dalbergia odorifera*, *Elaeocarpus decipiens*, and *Aquilaria sinensis*, respectively.

To visualize and excavate the relationships among different flower species, hierarchical clustering analysis and a heat map were performed based on the content of volatile compounds detected by the GC × GC-QTOFMS method (**Figure 2**). The heat map graphically represented the differences in volatile abundance using a color scale, with the change from pink to green indicating a ranging from low to high content. The result showed that flowers of the same species were closely grouped, even when harvested at different times. According to HCA, the three flower species were divided into two groups. Base on the colors corresponding to volatile contents, DO and AS were classified into one group due to their similar volatile content, including myrtenol, 3,4-dimethylbenzene, nerol, *etc*. In contrast, the contents of the main compounds in ED were distinct from those in the other two species, particularly aldehydes like (*E*)-3-hexenal, decanal, and (*E*)-4-nonenal, *etc*. Thus, ED was categorized into the second group.

### 3.5 GC-EAD and EAG responses

**Figure 3:**
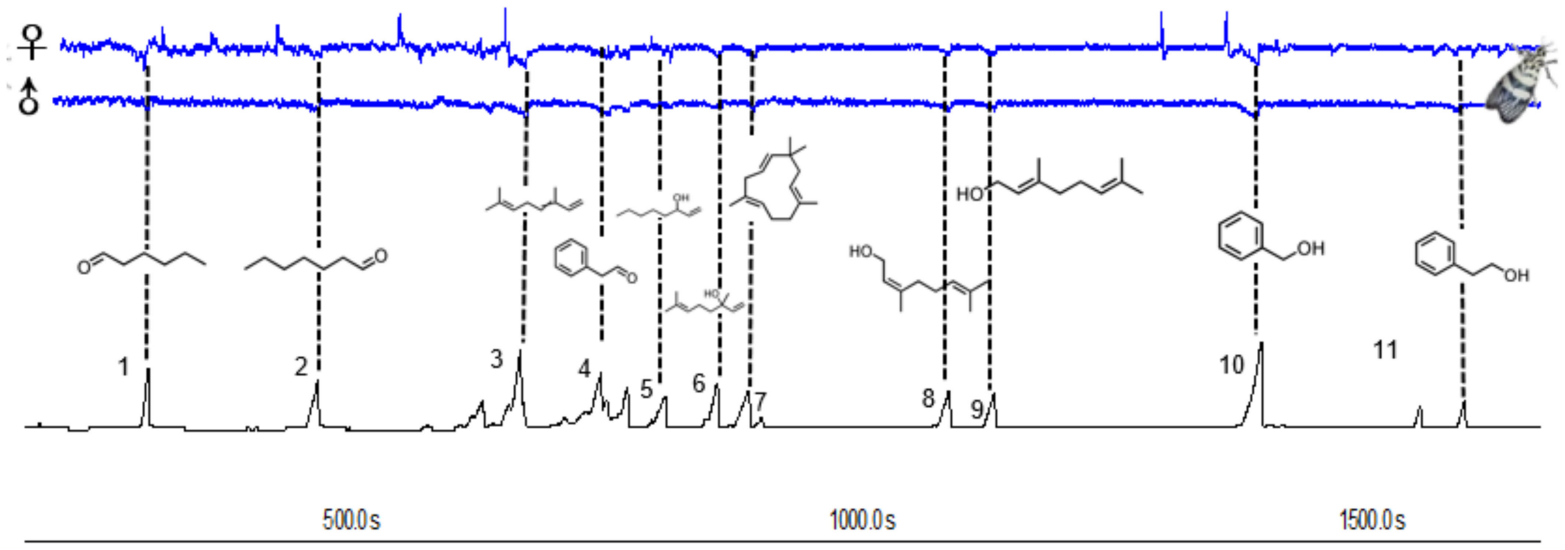
Responses of *Heortia vitessoides* female and male to volatiles detected by GC–EAD. The consistently EAD-active compounds in common floral volatile compounds were as follows: hexanal (1), heptanal (2), (*E*)-β-ocimene (3), benzaldehyde (4), 1-octen-3-ol (5), linalool (6), caryophyllene (7), nerol (8), geraniol (9), benzyl alcohol (10), phenylethyl alcohol (11) respectively.

The main common compounds were selected from the volatile components for electrophysiological testing. GC-EAD analyses revealed that the female and male antennae of *H. vitessoides* moths responded to all 11 compounds (**Figure 3**), which included 5 terpenoids, 3 alcohols, and 3 aldehydes, indicating that only a fraction of components were involved in the interaction between host/non-host flowers and the moths.

**Figure 4:**
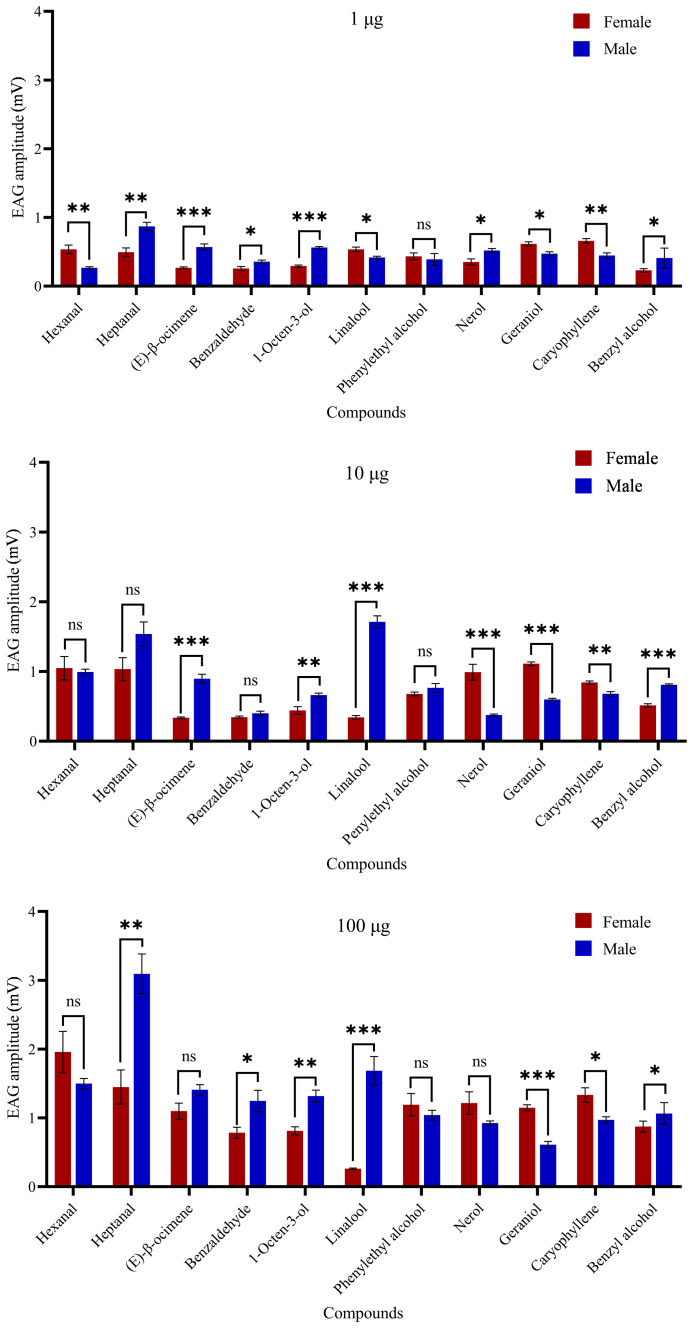
Electrophysiological response (means ± SE) values of *Heortia vitessoides* adult male and female moths to 11 floral volatiles at three different doses (1, 10, and 100 µg/µl). Significant differences between male and female EAG responses were analyzed by independent samples t-test (ns: not significant; *: 0.01 ≤ *p* < 0.05; **: 0.001 ≤ *p* < 0.01; ***: *p* < 0.001). N = 5 antennae per mean.

In electroantennogram test, the EAG responses of *H. vitessoides* moths to most volatiles were dose-dependent, with higher quantities triggering stronger responses. However, the response to benzyl alcohol appeared weaker with increasing dosage (**Figure 4**). Heptanal and hexanal elicited the largest EAG responses in males and females, respectively (**Figure 4**). Female moths appeared to be less sensitive to linalool than male moths (**Supplementary Table 4**; *P* < 0.05). Conversely, within each dose level, female antennal responses were significantly greater than males to geraniol and caryophyllene (*P* < 0.05). Thus, among the 11 chemical compounds tested, *H. vitessoides* moths only responded to certain FVOCs emitted by host/non-host plants.

### 3.6 Olfactometry bioassays

**Figure 5:**
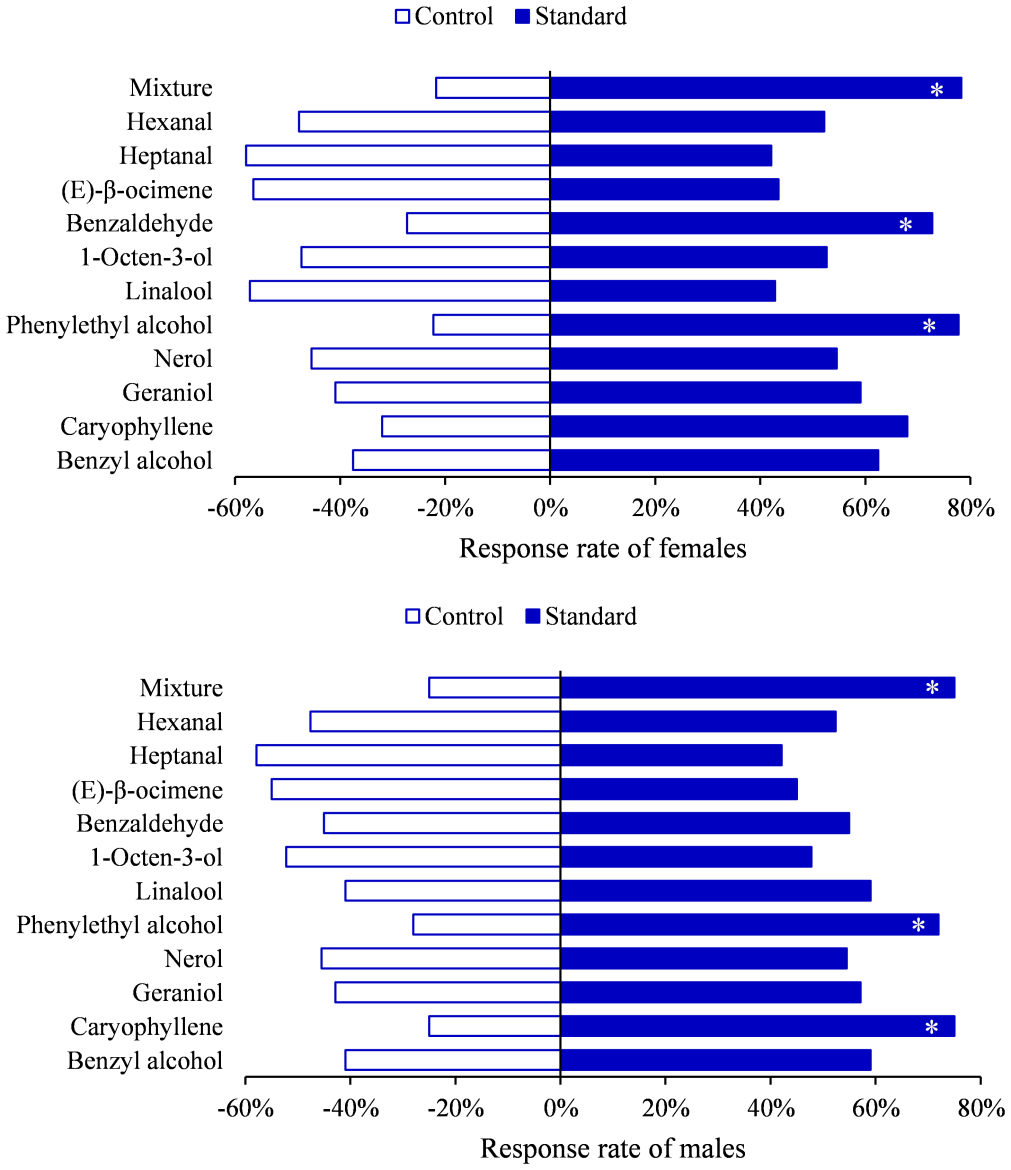
Behavioral responses of *Heortia vitessoides* to each compound tested in Y-tube olfactometer. (*: 0.01 ≤ *p* < 0.05).

To further demonstrate the biological activity of these compounds, Y tube choice experiments were conducted on *H. vitessoides* moths. Behavior bioassays using selected standards as the odor source showed that both genders were attracted to phenylethyl alcohol (female, *χ* ^2 =^ 5.5556, df=1, *P*=0.0184; male, *χ* ^2 =^ 4.8400, df=1, *P*=0.0278) or mixture (female, *χ* ^2 =^ 7.3478, df=1, *P*=0.0067; male, *χ* ^2 =^ 6.0000, df=1, *P*=0.0143) (**Figure 5**). Additionally, compared to hexane, females significantly preferred benzaldehyde (*χ* ^2 =^ 4.5455, df=1, *P*=0.0330), while males were more attracted to caryophyllene (*χ* ^2 =^ 6.0000, df=1, *P*=0.0143), and no responses were observed for other compounds (**Supplementary Table 5**; *P* > 0.05). Hence, *H. vitessoides* moths exhibit different sensitivity and selectivity to particular volatiles. Previous research has indicated that phenylpropanoid/benzenoid compounds (e.g., phenethyl alcohol) and terpenes (e.g., linalool, caryophyllene) were known to attract lepidopterous insects and have positive effects on insect attraction (Andersson et al., 2002). The moths’ preferences for specific volatile compounds potentially aid the pollinators in quickly locating valuable floral resources.

## 4 Conclusion

This study optimized an HS-SPME method for extracting volatiles from three flowers using single-factor optimization experiments. The optimal conditions were determined to be a sample quality of 125 mg, 45°C incubation temperature, 20 min equilibration time, and 40 min adsorption time. The current sampling method may not fully capture the profile of floral volatile components as released by the actual flowers, future research will focus on sampling blooming flowers *in situ* to accurately compare and understand the differences in volatile profiles. The floral scent profile of the host (AS) and non-host (ED, and DO) plants were then determined by GC×GC-QTOF-MS, revealing those aldehydes and alcohols as the two most abundant groups. OPLS-DA analysis showed a good fit of the mode and predictive ability, with samples harvested at different time periods being highly distinct groups. HCA divided flowers from the three species into two groups. Eleven compounds elicited responses in the *H. vitessoides* antenna through GC-EAD recordings, and in the Y-tube experiment, phenylethyl alcohol and a mixture of EAD-activate compounds were found to be attractive to both males and females. These results might provide candidate attractants based on FVOCs to monitor and control the *H. vitessoides* populations.

## Data Availability

The original contributions presented in the study are included in the article/**Supplementary Material**. Further inquiries can be directed to the corresponding author.
