# Supplementary material for: Quantitative analysis and characterization of floral volatiles, and the role of active compounds on the behavior of Heortia vitessoides
Source: Front Plant Sci. 2024 Aug 23;15:1439087. doi: 10.3389/fpls.2024.1439087 (PMC11377291; doi:10.3389/fpls.2024.1439087)
Supplement: Supplementary file 1 [file DataSheet1.pdf]

Supporting Information

Quantitative analysis and characterization of floral volatiles, and the role of active compounds on the behavior of the *Heortia vitessoides*

Chenyu Qian, Wenqi Xie, Zhongqi Su, Xiujun Wen, Tao Ma

College of Forestry and Landscape Architecture, South China Agricultural University, Guangzhou 510642, China

Corresponding author

E-mail: matao@scau.edu.cn (Tao Ma)

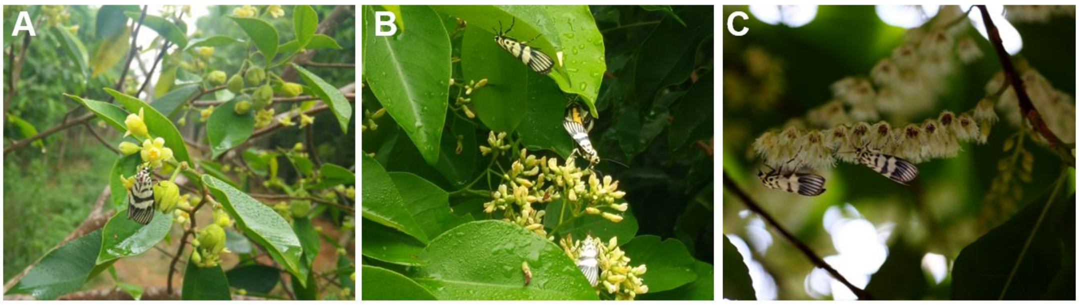

Figure S1 *Heortia vitessoides* moths are attracted by flowers of *Aquilaria sinensis* (A), *Dalbergia odorifera* (B), and *Elaeocarpus decipiens* (C).

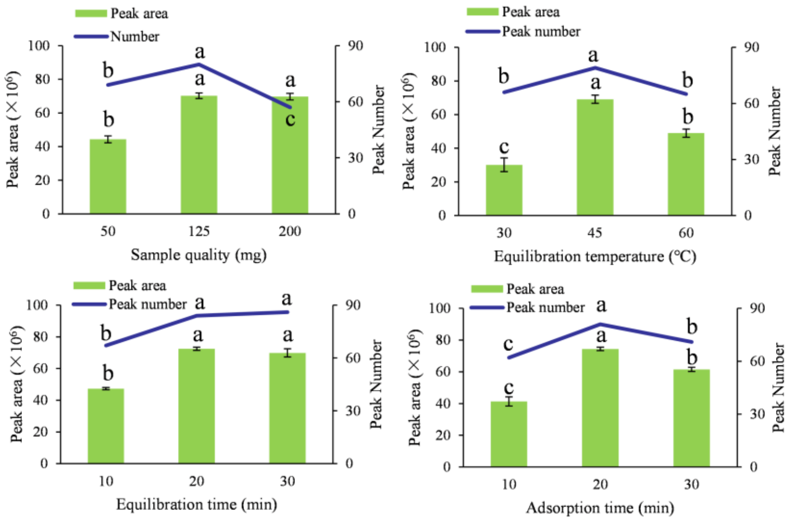

Figure S2 Extraction performance by different SPME parameters: sample quantity (B) incubation temperature (C), equilibration time (D), and adsorption time (E).

Table S1 Phenotypic traits of the three flower species.

Abbreviation	Latin name	Plant shape	Flower color	Inflorescence	Flower diameter (mm)	Flower season	Appearance
AS	*Aquilaria sinensis*	Macrophanerophytes	Green to yellow	Umbel	10±2	Spring	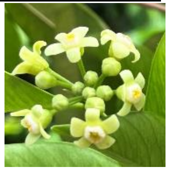
DO	*Dalbergia odorifera*	Macrophanerophytes	Yellow to white	Panicle	5±2	Spring	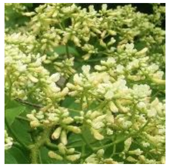
ED	*Elaeocarpus decipiens*	Macrophanerophytes	White	Raceme	40±5	Spring	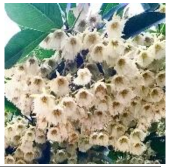

Table S2 Analytical precisions of standard compounds.

Compound	CAS	R^2	LOD (ng/mg)	LOQ (ng/mg)	RSD (%)
Hexanal	66-25-1	0.9983	0.034	0.115	2.805
Heptanal	111-71-7	0.9993	0.073	0.243	4.948
(*E*)-β-Ocimene	13877-91-3	0.9993	0.032	0.106	12.213
2-Hexenal	505-57-7	0.9997	0.032	0.107	8.494
1-Octen-3-ol	3391-86-4	0.9952	0.053	0.176	2.758
3-Octanol	589-98-0	0.9986	0.027	0.092	3.943
Nonanal	124-19-6	0.9967	0.164	0.547	0.690
Benzaldehyde	100-52-7	0.9979	0.084	0.280	11.233
Linalool	78-70-6	0.9992	0.026	0.087	2.486
Caryophyllene	87-44-5	0.9988	0.018	0.06	1.841
Phenylacetaldehyde	122-78-1	0.9992	0.026	0.088	11.394
Nerol	106-25-2	0.9974	0.036	0.121	1.803
Geraniol	106-24-1	0.9976	0.035	0.116	6.202
Benzyl alcohol	100-51-6	0.9992	0.018	0.061	6.729
Phenylethyl Alcohol	60-12-8	0.9990	0.194	0.647	4.985

Table S3 Major volatile compounds extracted from three flower species at 2 periods and analyzed by GC×GC–QTOFMS (ng/mg), the peaks were identified based on their RT (Kovats index) and MS spectra.

Compounds	Peak I (min)[a]	Peak II (s)[a]	*Aquilaria sinensis*		*Dalbergia odorifera*		*Elaeocarpus decipiens*		Matching Factor		RI_{exp}[b]	RI_{lib}[c]
			Day	Night	Day	Night	Day	Night	Forward	Reverse		
**Aldehyde**												
Hexanal	6.7657	2.261	5.678±0.374	6.664±0.933	7.94±0.182	7.135±1.024	4.442±0.556	3.025±0.126	900	900	-	1083-P
(*E*)-3-Hexenal	8.1652	1.365	-	-	-	-	0.126±0.011	0.088±0.021	828	857	1145	1146-P
2-Methylidenehexanal	8.4653	2.507	0.868±0.028	1.029±0.146	-	0.604±0.014	-	0.531±0.026	900	900	1159	1160-P
Heptanal	9.1654	2.564	5.266±0.307	5.618±0.43	3.411±0.237	4.511±0.207	5.003±1.314	6.576±0.306	900	900	1189	1184-P
2-Hexenal	9.9655	2.227	0.494±0.006	0.607±0.099	0.823±0.033	0.852±0.114	1.228±0.057	1.234±0.073	900	900	1221	1213-P
(*E*)-2-Hexenal	10.0652	1.48	0.119±0.014	0.5±0.07	-	-	0.197±0.015	0.125±0.013	900	900	1224	1216-P
(*Z*)-4-Heptenal	10.6657	2.315	-	-	0.327±0.024	0.471±0.022	-	-	900	900	1248	1240-P
Octanal	11.7648	2.813	1.004±0.007	2.028±0.046	-	-	0.675±0.007	0.986±0.077	900	900	1290	1289-P
(*Z*)-2-Heptenal	12.6653	2.387	-	-	0.779±0.056	1.146±0.105	-	-	900	900	1325	1322-P
Benzaldehyde	12.7654	1.902	2.739±0.116	4.167±0.325	0.751±0.011	3.221±0.113	4.052±0.187	6.842±0.15	900	900	1329	1330-P
Nonanal	14.4656	2.86	4.112±0.557	5.831±0.716	0.336±0.002	0.57±0.035	3.75±0.158	3.134±0.058	900	900	1394	1394-P
(*E*,*E*)-2,4-Hexadienal	14.7655	2.014	-	-	-	-	0.139±0.01	0.444±0.112	900	900	1405	1400-P
(*E*)-2-Octenal	15.3657	2.413	0.071±0.007	0.122±0.019	0.329±0.013	0.442±0.016	0.555±0.206	0.205±0.111	900	900	1430	1429-P
(*E*)-4-Nonenal	15.6657	2.536	-	-	-	-	0.16±0.01	0.271±0.077	828	873	1442	1435-P
(*E*)-6-Nonenal	15.9651	2.642	-	-	0.385±0.007	0.106±0.054	-	-	900	900	1454	1438-P
(*E*, *E*)-2,4-Heptadienal	16.9653	2.098	-	-	0.392±0.026	0.677±0.128	-	-	900	900	1494	1495-P
Decanal	17.165	2.969	-	0.118±0.004	-	-	0.245±0.034	0.64±0.051	755	854	1502	1498-P
(*E*)-2-Nonenal	17.9654	2.519	0.36±0.042	0.463±0.035	-	0.959±0.018	1.672±0.093	4.712±0.037	900	900	1526	1520-P
Phenylacetaldehyde	20.7652	1.76	0.782±0.375	0.743±0.055	0.173±0.008	0.612±0.065	0.357±0.022	0.7±0.068	871	879	1645	1640-P
(*E*)-2-Butyl-2-octenal	21.4652	1.814	0.058±0.005	0.078±0.017	0.296±0.005	-	0.08±0.005	-	900	900	1672	1656-P

3,4-Dimethylbenzaldehyde	24.2656	2.146	-	-	0.127±0.005	-	-	0.354±0.056	875	878	1807	1790-P
**Alcohols**												
1-Penten-3-ol	8.4653	1.581	0.156±0.02	0.653±0.758	0.722±0.028	0.95±0.092	0.361±0.016	0.181±0.028	900	900	1158	1159-P
2-Hexyn-1-ol	9.5651	2.382	0.253±0.067	0.233±0.019	-	-	-	-	900	900	1206	1207-P
1-Pentanol	10.6657	1.617	0.344±0.040	-	0.132±0.001	0.342±0.004	-	-	900	900	1247	1250-P
(*Z*)-2-Pentenol	12.5653	1.541	0.7±0.028	0.659±0.036	2.402±0.077	3.073±0.066	1.047±0.117	1.173±0.132	900	900	1320	1318-P
1-Hexanol	13.3654	1.687	-	4.213±0.293	3.143±0.088	-	1.838±0.027	1.66±0.03	900	900	1351	1355-P
2-Methyl-4enten-1-ol	13.6654	1.639	0.287±0.101	0.359±0.023	0.128±0.006	0.223±0.043	-	0.054±0.005	900	900	1363	1375-P
3-Octanol	14.4656	1.933	0.146±0.01	0.117±0.008	0.984±0.052	1.121±0.08	4.256±0.187	5.585±0.101	900	900	1394	1393-P
(*E*)-2-Hexen-1-ol	14.7653	1.626	-	-	-	-	0.065±0.016	0.529±0.084	762	804	1405	1405-P
1-Octen-3-ol	14.8658	1.731	0.924±0.053	0.977±0.091	1.549±0.406	2.037±0.008	0.306±0.07	0.083±0.018	867	867	1449	1450-P
2-Ethyl-1-hexanol	16.7652	1.701	0.082±0.01	0.248±0.006	-	0.761±0.038	0.156±0.004	0.131±0.001	900	900	1485	1491-P
1-Octanol	18.4655	1.788	1.192±0.041	1.449±0.075	0.734±0.07	0.876±0.032	0.241±0.016	0.703±0.073	900	900	1547	1547-P
1,3-Dimethylcyclohexanol	19.8657	1.671	-	-	0.369±0.007	0.605±0.056	-	-	900	900	1606	1611-P
(*E*)-2-Octen-1-ol	20.0658	2.729	-	-	-	0.597±0.054	0.072±0.005	-	800	831	1614	1614-P
1-Nonanol	20.8651	1.904	-	-	-	-	0.156±0.005	1.587±0.018	900	900	1658	1660-P
1-Nonen-4-ol	21.1653	3.061	-	-	0.125±0.024	1.046±0.054	-	-	900	900	1654	1655
(*Z*)-3-Nonen-1-ol	21.7654	2.114	5.999±0.069	6.689±0.702	3.103±0.047	3.694±0.064	0.651±0.071	1.496±0.062	900	900	1684	1682-P
(*Z*)-6-Nonen-1-ol	22.5651	2.058	0.59±0.027	-	-	0.824±0.052	-	-	718	782	1714	1711-P
(*E*,*Z*)-3,6-Nonadien-1-ol	23.1656	1.691	-	-	-	-	0.119±0.003	0.284±0.024	815	824	1749	1747-P
Benzyl alcohol	23.2564	2.45	10.277±0.193	8.85±0.141	2.261±0.115	1.15±0.138	0.771±0.004	2.948±2.111	900	900	1768	1770-P
Phenylethyl Alcohol	26.7655	1.621	0.313±0.037	0.165±0.024	1.384±0.076	0.606±0.034	0.685±0.016	4.948±4.111	900	900	1907	1906-P
**Terpenoids**												
(*E*)-β-Ocimene	12.0227	3.295	0.096±0.004	0.779±0.025	0.231±0.023	0.587±0.038	0.784±0.026	0.6±0.061	900	900	1252	1250-P
4,8-Dimethyl-3,7-nonadien-2-ol	12.2651	3.617	1.256±0.062	-	-	-	-	-	877	826	1310	1311-P
6-Methyl-5-hepten-2-one	13.0653	2.437	0.699±0.026	0.672±0.08	2.344±0.319	2.545±0.321	0.622±0.033	0.273±0.06	900	900	1340	1338-P

6-Methyl-5-hepten-2-ol	16.1652	1.822	-	-	0.216±0.014	0.311±0.011	-	-	900	900	1461	1465-P
Linalool	15.1765	1.925	2.311±0.101	2.059±0.241	0.95±0.087	1.149±0.173	0.355±0.016	0.788±0.012	900	900	1465	1464-P
Caryophyllene	15.7122	4.514	1.341±0.035	3.094±0.011	1.549±0.011	2.34±0.061	2.097±0.001	2.079±0.003	900	900	1489	1495-P
Nerol	18.3247	2.154	0.784±0.033	0.392±0.081	0.34±0.006	0.464±0.02	0.116±0.001	0.752±0.07	900	900	1537	1538-P
Geraniol	19.1652	2.712	4.48±0.031	4.213±0.415	3.143±0.125	3.596±0.095	0.684±0.067	0.948±0.111	871	873	1565	1567-P
9-Epi-caryophyllene	19.2656	2.282	0.295±0.02	0.192±0.019	1.05±0.049	1.14±0.062	0.396±0.005	2.511±0.155	900	900	1578	1572-P
β-Gurjunene	19.3653	5.009	-	0.183±0.005	-	-	-	-	796	799	1595	1605-P
β-Elemen	19.3655	3.872	0.019±0.005	-	-	-	-	-	814	873	1595	1591-P
Bornyl acetate	19.1653	3.277	-	0.117±0.001	-	-	-	-	886	888	1586	1581-P
β-Cyclocitral	20.1658	2.064	0.316±0.072	0.169±0.007		1.012±0.073	0.077±0.001	-	900	900	1624	1611-P
4-Oxoisophorone	22.1654	1.714	0.435±0.203	-	0.217±0.002	0.286±0.135	2.979±0.081	11.26±0.219	900	900	1697	1676-P
α-Farnesene	22.9655	1.704	-	0.745±0.059	-	-	-	-	900	900	1751	1746-P
Myrtenol	24.0657	1.76	-	-	-	-	0.305±0.024	0.504±0.232	849	853	1789	1796-P
Geranyl acetone	25.2651	2.723	-	-	0.136±0.006	4.451±0.14	0.073±0.013	1.948±1.111	900	900	1855	1859-P
β-Ionone	28.0657	2.4	0.054±0.005	-	0.217±0.023	1.515±1.025	0.031±0.005	5.948±5.111	900	900	1938	1940-P
**Heterocyclic compounds**												
2-Pentylfuran	10.3656	2.836	0.033±0.008	-	0.876±0.087	1.067±0.127	3.091±0.232	3.044±0.172	900	900	1237	1231-P
(*Z*)-2-(2-pentenyl)furan	12.1652	2.634	-	-	0.259±0.017	0.543±0.102	1.298±0.048	1.191±0.006	900	900	1306	1305-P
(*E*)-Linalool 3,7-oxide	22.9652	3.575	0.225±0.038	0.644±0.046	-	0.219±0.009	0.138±0.002	-	871	875	1474	1452-P
(*Z*)-Linalool 3,7-oxide	23.5653	2.113	-	-	0.19±0.007	-	0.65±0.05	0.438±0.123	858	865	1446	1445-P
**Ketone**												
Cyclohexanone	11.7651	2.501	-	-	1.224±0.047	2.486±0.328	-	-	857	872	1290	1291-P
3-Octanone	14.4654	1.966	0.275±0.055	-	0.729±0.03	2.942±0.186	-	-	900	900	1394	1393-P
3-Octen-2-one	14.7656	2.485	-	-	0.295±0.038	0.448±0.028	-	-	900	900	1406	1411-P
(*E*,*E*)-3,5-Octadien-2-one	18.9656	4.52	-	-	0.017±0.003	0.043±0.008	-	-	900	900	1568	1570-P
3,5,5-Trimethylcyclohexane-1,4-dione	23.8657	1.809	-	-	0.199±0.007	-	2.566±0.032	3.139±0.686	900	900	1780	1778-P

**Ester**												
(*E*)-4-Hexenyl acetate	12.5651	2.631	0.149±0.007	0.322±0.007	-	-	-	-	873	885	1321	1326-P
Methyl benzoate	20.465	3.07	4.128±0.405	2.574±0.24	0.213±0.005	0.483±0.011	-	-	900	900	1628	1612-P
Benzyl acetate	22.6655	2.28	-	0.241±0.016	-	-	-	-	900	900	1732	1720-P
(Z)-3-Hexenyl benzoate	30.6654	3.219	0.507±0.024	0.739±0.074	-	2.515±2.025	-	-	900	900	2124	2126-P
**Aromatic Hydrocarbon**												
1-Ethyl-2,4-dimethylbenzene	13.7653	3.143	-	-	-	-	-	0.201±0.033	895	899	1368	1348-P
1,2,3,5-Tetramethylbenzene	15.1657	3	-	-	0.269±0.029	0.428±0.037	-	0.604±0.065	870	879	1422	1422-P
1,3-Di-tert-butylbenzene	15.3657	4.093	0.388±0.041	0.511±0.022	-	0.057±0.012	0.27±0.02	0.152±0.11	851	885	1431	1427-P
1,3-Diethyl-4-methyl-benzene	15.7656	3.317	-	-	-	-	-	0.387±0.005	793	806	1446	1440-P
4-Ethylstyrene	16.4652	3.055	-	-	0.139±0.008	0.125±0.027	-	0.148±0.018	900	900	1474	1462-P
1,2,3,4-Tetramethylbenzene	16.7651	3.009	-	-	0.143±0.004	0.264±0.022	-	0.482±0.037	879	879	1486	1462-P
1-Ethyl-3-isopropylbenzene	17.5652	3.159	-	-	-	-	-	7.651±0.629	865	870	1519	1520-P
Pentamethylbenzene	20.5652	1.911	-	-	-	-	-	0.487±0.065	890	892	1642	1656-P
Azulene	22.7655	1.802	-	-	0.198±0.003	-	-	1.159±0.061	919	919	1737	1729-P
α-Methylnaphthalene	26.3654	1.517	-	-	-	2.973±0.07	-	0.179±0.111	890	896	1878	1884-P
**Other**												
(*E*)-3-Ethyl-2-methyl-1,3-hexadiene	15.0657	2.529	-	0.617±0.024	0.121±0.008	0.217±0.012	0.36±0.015	0.425±0.034	900	900	1418	1413-P
Methyl salicylate	23.6657	1.981	-	0.245±0.008	-	1.05±0.011	-	-	888	906	1776	1765-P
Benzyl nitrile	26.9655	2.657	-	-	0.129±0.013	0.515±0.025	-	-	900	900	1927	1909-P
Methyleugenol	30.6651	2.414	-	-	-	-	0.161±0.003	-	809	825	2007	2013-P

[a] Retention time calculated on DB-Wax and DB-17capillary column.

[b] Retention indices from the present study measured according to C_8-C_{25} alkanes on a DB-Wax column.

capillary column

[c] Retention indices from https://webbook.nist.gov/chemistry

"-" means not detected or below the limits of detection.

Table S4 Statistical results of GC-EAD responses of *Heortia vitessoides* females and males to standards at different dosages. (Independent sample *t*-test, *P*<0.05).

Standards	loading (μg)	t-test
Hexanal	1	t=4.26, df=8, P=0.002
Heptanal	1	t=-4.21, df=8, P=0.003
(*E*)-β-Ocimene	1	t=-6.45, df=8, P=0.000
Benzaldehyde	1	t=-2.58, df=8, P=0.033
1-Octen-3-ol	1	t=-12.82, df=8, P<0.000
Linalool	1	t=3.16, df=8, P=0.013
Phenylethyl alcohol	1	t=0.44, df=8, P=0.672
Nerol	1	t=-3.1, df=8, P=0.015
Geraniol	1	t=3.25, df=8, P=0.012
Caryophyllene	1	t=4.04, df=8, P=0.004
Benzyl alcohol	1	t=-2.72, df=8, P=0.026
Hexanal	10	t=0.33, df=8, P=0.748
Heptanal	10	t=-2.09, df=8, P=0.070
(*E*)-β-Ocimene	10	t=-8.57, df=8, P<0.001
Benzaldehyde	10	t=-1.52, df=8, P=0.166
1-Octen-3-ol	10	t=-3.55, df=8, P=0.008
Linalool	10	t=-15.17, df=8, P<0.000
Phenylethyl alcohol	10	t=-1.28, df=8, P=0.236
Nerol	10	t=5.37, df=8, P=0.0007
Geraniol	10	t=15.8, df=8, P<0.0001
Caryophyllene	10	t=4.17, df=8, P=0.003
Benzyl alcohol	10	t=-10.55, df=8, P<0.000
Hexanal	100	t=1.49, df=8, P=0.175
Heptanal	100	t=-4.31, df=8, P=0.003
(*E*)-β-Ocimene	100	t=-2.2, df=8, P=0.059
Benzaldehyde	100	t=-2.66, df=8, P=0.029
1-Octen-3-ol	100	t=-4.8, df=8, P=0.001
Linalool	100	t=-6.84, df=8, P=0.0001
Phenylethyl alcohol	100	t=0.85, df=8, P=0.419
Nerol	100	t=1.75, df=8, P=0.118
Geraniol	100	t=8.31, df=8, P<0.000
Caryophyllene	100	t=3.12, df=8, P=0.014
Benzyl alcohol	100	t=-2.6, df=8, P=0.031

Table S5 Statistical analysis of the choice data for female and male *Heortia vitessoides* in Y-tube olfactometer bioassays with different standards against hexane (control).

Standards	Female response					Male response				
	control	Standards	NC*	Statistical results		control	Standards	NC	Statistical results	
				χ^2	P				χ^2	P
Mixture	5	18	7	7.348	0.007	6	18	6	6.000	0.014
Hexanal	11	12	7	0.044	0.835	10	11	9	0.048	0.827
Heptanal	11	8	11	0.474	0.491	11	8	11	0.474	0.491
(E)-β-ocimene	13	10	7	0.391	0.532	11	9	10	0.200	0.655
Benzaldehyde	6	16	8	4.546	0.033	9	11	10	0.200	0.655
1-Octen-3-ol	9	10	11	0.053	0.819	12	11	7	0.044	0.835
Linalool	12	9	9	0.429	0.513	9	13	8	0.727	0.394
Phenylethyl alcohol	4	14	12	5.556	0.018	7	18	5	4.840	0.028
Nerol	10	12	8	0.182	0.670	10	12	8	0.182	0.670
Geraniol	9	13	8	0.727	0.394	9	12	9	0.429	0.513
Caryophyllene	8	17	5	3.240	0.072	6	18	6	6.000	0.014
Benzyl alcohol	9	15	6	1.500	0.221	9	13	8	0.727	0.394

*NC: No choice.
